# Evaluation of three methods for biomass estimation in small invertebrates, using three large disparate parasite species as model organisms

**DOI:** 10.1038/s41598-018-22304-x

**Published:** 2018-03-01

**Authors:** Cristina Llopis-Belenguer, Isabel Blasco-Costa, Juan Antonio Balbuena

**Affiliations:** 1 Symbiosis Lab, Cavanilles Institute of Biodiversity and Evolutionary Biology, University of Valencia, PO Box 22085, 46071 Valencia, Spain; 2 Natural History Museum of Geneva, PO Box 6434, CH-1211 Geneva 6, Switzerland

## Abstract

Invertebrate biomass is considered one of the main factors driving processes in ecosystems. It can be measured directly, primarily by weighing individuals, but more often indirect estimators are used. We developed two indirect and non-destructive approaches to estimate biomass of small invertebrates in a simple manner. The first one was based on clay modelling and the second one was based on image analysis implemented with open-source software. Furthermore, we tested the accuracy of the widely used geometric approximation method (third method). We applied these three different methods to three morphologically disparate model species, an acanthocephalan worm, a crustacean and a flatworm. To validate our indirect estimations and to test their accuracy, we weighed specimens of the three species and calculated their tissue densities. Additionally, we propose an uncomplicated technique to estimate thickness of individuals under a microscope, a required measurement for two of the three indirect methods tested. The indirect methods proposed in this paper provided the best approximation to direct measurements. Despite its wide use, the geometric approximation method showed the lowest accuracy. The approaches developed herein are timely because the recently increasing number of studies requiring reliable biomass estimates for small invertebrates to explain crucial processes in ecosystems.

## Introduction

Biomass is the mass of living organisms from a given area or ecosystem at a point in time that can be found in liquid, gas and solid forms^1^. In ecology, the importance of quantifying biomass stems from understanding the processes that drive changes in ecosystems^2^. For instance, vegetal biomass has been considered as the principal factor promoting the first phase of ecological succession in forests^2^ high loads of soil microbial biomass reduces the efficacy of a biological agent on plant pathogens^3^ species with greater biomass are expected to have lower probabilities to become extinct, which might reduce the impact on ecosystem functioning under extinction scenarios^4^ and the variation in abundance (as proxy of biomass), not in richness, in few species of bees drive ecosystem services^5^. Furthermore, it has been suggested that measuring diversity using biomass in community ecology studies might be more insightful than using species abundances^6^. Although biomass is an extremely important attribute, its estimation represents often a challenge, among other reasons, because of the difficulty in identifying the unit measured^7^, the need to manipulate or destruct samples^8^, the lack of resolution in large-scale studies^9,10^ or the impossibility to discern dead from alive individuals^11^.

Invertebrates are often the cornerstone of ecosystems^12,13^ and recent studies have shown that their biomass is greater than that previously thought^14,15^. Different methods have been proposed to study the allocation of biomass between various taxonomic groups of invertebrates, mostly arthropods, but also considered groups include sponges, cnidarians, platyhelminths, annelids, acanthocephalans, nematodes, molluscs, nemerteans, echinoderms, bryozoans and urochordates have been considered^14,16–24^. As direct measurements of biomass of small invertebrates (i.e. body length from µm to a few mm), common practices include weighing wet^8,13,25^, dry^26^ or ash-free dry masses^27^; and measuring elements or biomolecules in a sample^13,19^. However, small body size and high abundance often hampers direct quantification of biomass in many organisms^20^. Therefore, indirect estimators have been proposed, such as using body surface areas or volumes as proxies of individual mass based on linear measurements^17,18,28–35^, linear lengths of different features converted into biomass through generalised regression equations^20–24,36^, displacement of water volume in a graduated cylinder^8,13,28,32,37^, or biovolume estimated using confocal microscopy and image analysis^38^. Nonetheless, most of these methods are taxon- or age-specific, destructive, laborious and time consuming or overlook the contribution of appendages to the total individual mass.

**Figure 1:**
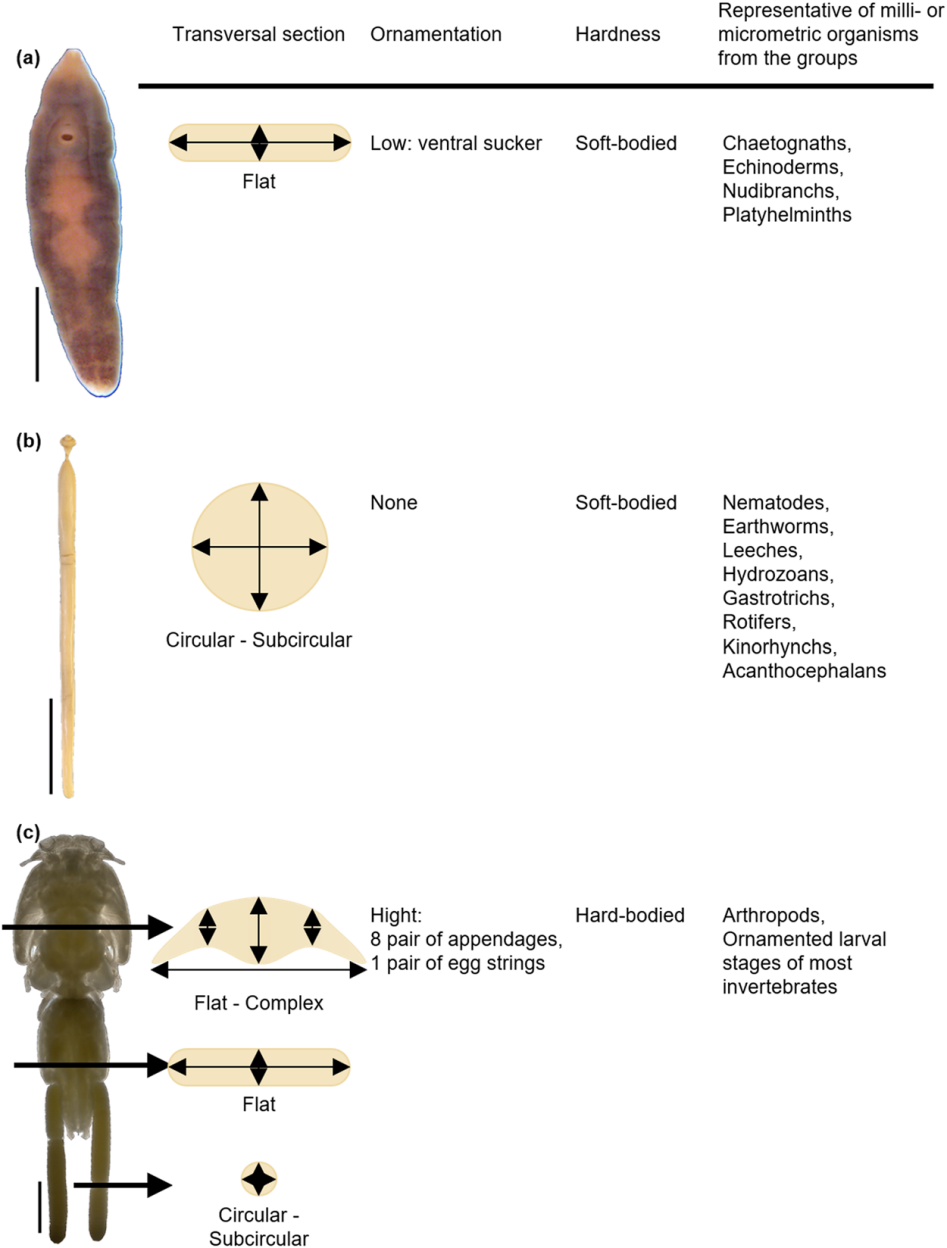
Phenotypic traits that justify the use of the model species. (**a**) *Campula oblonga*, (**b**) *Bolbosoma capitatum* and (**c**) *Caligus elongatus* as a model species of the biomass indirect estimation methods. Scale bars 0.5 mm, 2 cm and 5 cm respectively.

In the present paper, we evaluate three different approaches to estimate biomass in small invertebrates, using three notably dissimilar in shape parasite species (an acanthocephalan, a crustacean and a flatworm) as model organisms. Although neglected at first (see references in: Lagrue & Poulin)^35^, the increasing number of studies pointing at the importance of parasite biomass in ecosystem functioning^17,30,32,35,39–41^ demand accurate and easy-to-apply procedures to estimate this component of biodiversity. We contend that, although the three model species analysed here, each have a parasitic mode of life, they are good representatives of morphological diversity of small invertebrates in general, because they represent three different phyla, cover both soft and hard-body species, with different transversal sections and levels of ornamentation (Fig. 1).

Despite the wide use of linear measurements to implement geometric approximations (see references above), to the best of our knowledge, their accuracy has not been validated with alternative methods for size/biomass estimators before. Due to the growing number of studies testing functions of both free-living small invertebrates (e.g.)^12,14^ and parasites (e.g.)^35^ in ecosystems this real critical appraisal is long overdue. Using three phylogenetic and morphologically disparate invertebrate species as models, the aim of our work was twofold: (i) to develop and evaluate two non-destructive approaches to indirectly estimate individual mass, which can be applied to a wide range of small invertebrate and entails the challenge of being applicable to a huge diversity of forms; and (ii) to test the accuracy of these two methods in comparison with direct estimation and the approaches based on geometric approximations widely used in previous studies.

## Material and Methods

### Model Specimens

We based our analyses on three disparate species (Fig. 1): the flatworm *Campula oblonga* Cobblod (Platyhelminthes, Trematoda). These were selected as models because of their relatively large size (mm) and their availability in sufficient numbers for the present study, thereby allowing to estimate their biomass directly. *C. oblonga* is a relatively large trematode (4–8 mm long × 1–2 mm wide), which inhabits the hepatic and bile ducts of small toothed whales (mostly Phocoenidae) in the northern hemisphere^42^. *B. capitatum* is a large acanthocephalan (34–99 mm × 1.5–3.5 mm) found in the intestine of large, pelagic toothed whales all over the world^43^. *Ca. elongatus* (body length 5–6 mm) is an extremely common parasitic copepod in the North Atlantic, which has been reported on over 80 species of teleosts and elasmobranchs^44,45^.

The specimens used herein are part of our research institute parasite collection’s and have been collected over the years in necropsies of cetaceans and fishes. *Campula oblonga* individuals were collected from *Phocoena phocoena* (Linnaeus), *B*. *capitatum* from *Globicephala melas* and *Pseudorca crassidens* and *Ca. elongatus* from *Gadus morhua* Linnaeus. The parasite specimens were in good condition at the time of collection, i.e. no sign of degradation of lysis was observed, and either preserved in ethanol 70% (*B. capitatum, C. oblonga* and *Ca. elongatus*) or in microscope slides mounted in Canada balsam (*C. oblonga*). Since there is a marked sexual dimorphism in *Ca. elongatus*, the specimens used herein for the sake of demonstration of the methods were all females. The reader is referred to the Discussion for guidelines for dealing with intraspecific morphological differences.

**Figure 2:**
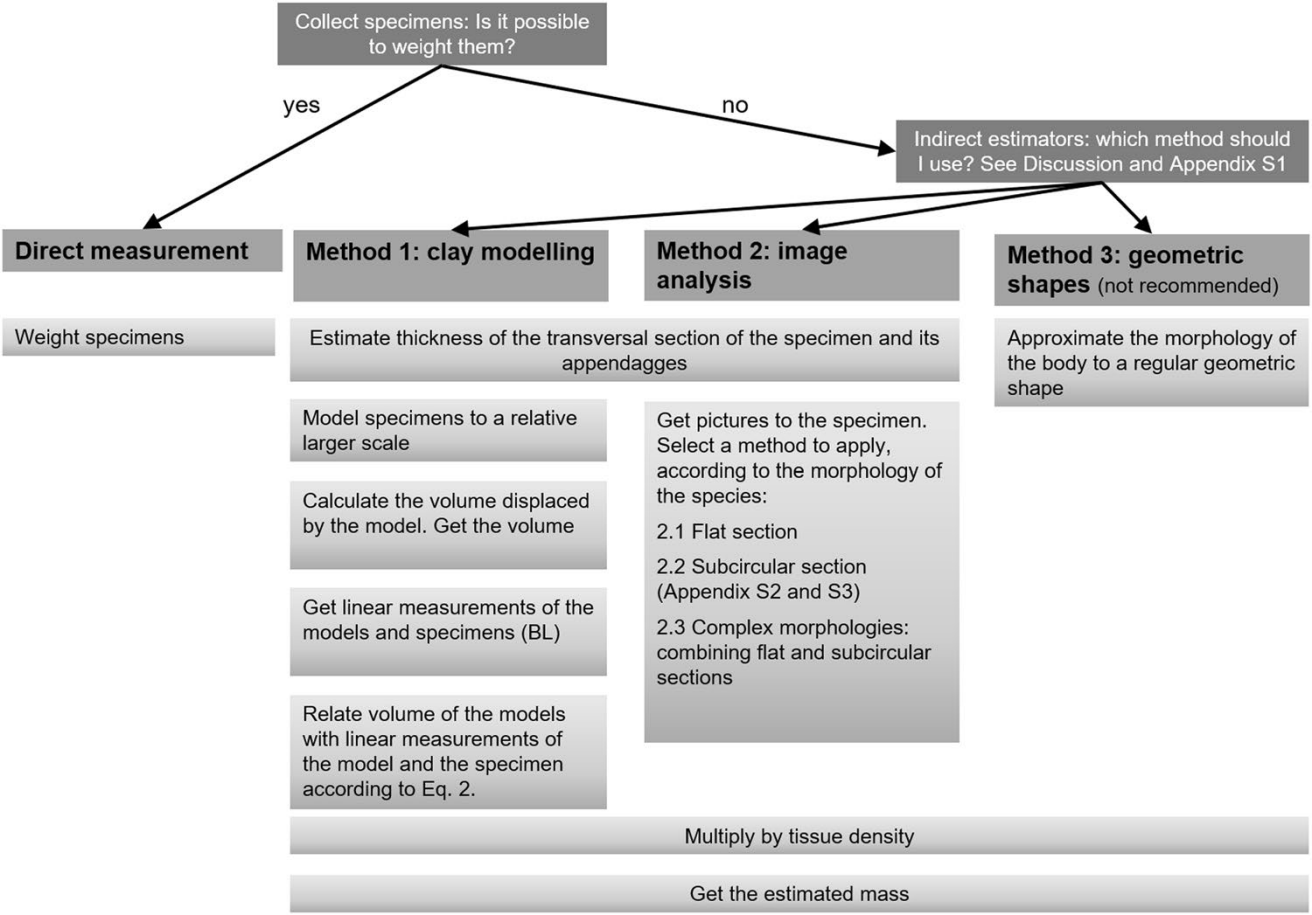
Flowchart summarising the steps of each method.

In this paper, we performed (1) direct measurements of mass of parasites; and indirect measurements based on (2) clay modelling, (3) image analysis (two approaches) and (4) approximation of the actual body shapes to regular geometric shapes. For direct measurements, in *C*. *oblonga* we weighed a group of 41 individuals to calculate the mean individual body mass; whereas in *B*. *capitatum* and *Ca. elongatus* we weighed 20 specimens of each species individually to measure individual weights. For indirect measurements, we estimated first body volume. In *C*. *oblonga*, we used 20 individuals mounted on permanent slides; whereas in *B*. *capitatum* and *Ca. elongatus*, we used the same 20 individuals each mentioned above. Then, we multiplied body volume by tissue density estimated for each species to estimate individual body mass for each indirect approximation. A flowchart of the process is given in Fig. 2.

### Direct Estimation

Direct biomass estimates of the specimens were used as a benchmark for the other methods, enabling quantifying the error associated with indirect methods. Mean biomass was estimated using the method described in the literature^16^. Since specimens had been stored in 70° ethanol for 10–30 years, they were placed in 0.9% saline solution (9 g NaCl per 1 L distillated water) for 1 to 5 days to allow the tissues to re-hydrate. Then mean individual mass was calculated as follows: the excess of water was removed by leaving the individuals briefly on blotting paper. For *C*. *oblonga*, we weighed two sets of 20 (*w*_*set1*_) and 21 (*w*_*set2*_) individuals from two different host individuals and calculated the mean individual weight ($\bar{w}$_*individual*_) as follows (Eq. 1):

1 $${\bar{w}}_{individual}=(({w}_{{set}{1}}/20)+({w}_{{set}{2}}/21))/2$$

For *B*. *capitatum* and *Ca. elongatus*, we chose 20 individuals of each species. *Bolbosoma capitatum* were collected from 12 and *Ca. elongatus* from 6 different host individuals. Individuals of both species were weighed individually. The specimens were weighed to the nearest milligram twice for each species.

Given that the indirect methods described herein are based on estimation of body volume, an estimate of tissue density is required for conversion to biomass. For this purpose, we weighed and measured the volume displaced in a graduated cylinder by a mass of new sets of several hundreds (*C*. *oblonga*) or tens (*B*. *capitatum* and *Ca. elongatus*) of re-hydrated specimens. We did these procedures twice and used the averaged quotient of mass to volume as density of each species.

### Thickness Estimation

The indirect methods presented here require expert predictions about the transversal section of specimens. In the simplest case, as in *B. capitatum*, it can be assumed to be subcircular (Fig. 1b) and, thus, thickness and width are expected to be nearly equivalent along the longitudinal axis.

In other cases, as in our flatworm or crustacean models, the transversal section is far from circular, which requires its modelling based on body thickness estimates (Fig. 1a and c). In the published descriptions, measurements of thickness are often not available^46^ as specimens are viewed and depicted frontally (dorso-ventrally rather than laterally). In the present study, the thickness of specimens in permanent mounts was measured individually under a microscope. First, we marked both sides of a microscope slide 100 µm thick, placed it under the microscope and focused on one of the sides. For a given magnification, we recorded the number of divisions of the micrometre knob taken to focus on the opposite side. This operation was repeated ten times and the mean number of divisions was used to establish the vertical displacement accounted by each knob division. Following the same approach, we measured the thickness of the specimens of *C. oblonga* mounted in Canada balsam on slides and *Ca. elongatus* mounted on non-permanent slides in saline solution at 20× and 10× magnification, respectively. For *C. oblonga*, body thickness was measured at the levels of pharynx, ventral sucker and posterior end of vitellarium. We also measured the thickness of the ventral sucker to improve the accuracy of our proposed method (Table S1). For *Ca. elongatus*, body thickness was measured at the lateral and central areas of the cephalothorax, fourth pedigerous somite, genital segment and abdomen. Additionally, we measured the thickness of one appendage of each of the 8 pairs occurring in adult specimens of *Ca. elongatus*: Antennae 1–2, maxillae, maxilliped and legs 1–4 (Tables S2 and S3).

### Indirect Method 1: Clay Modelling

**Figure 3:**
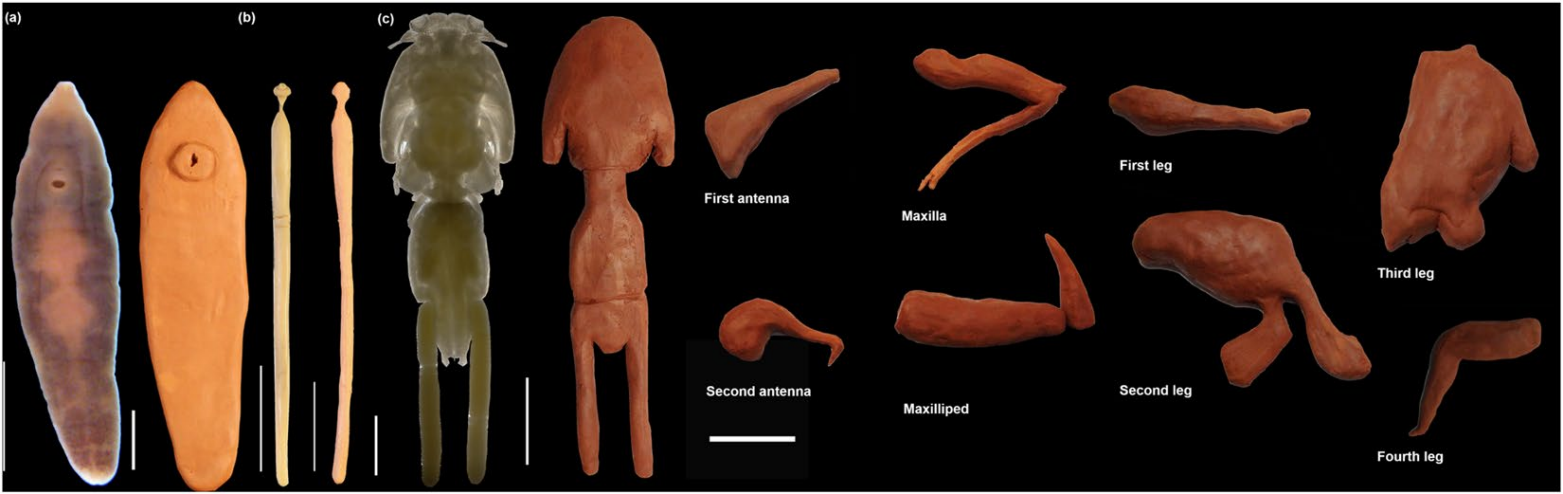
Specimens and clay figurines of the model species. (**a**) *Campula oblonga*, scale bars 0.5 mm and 2 cm respectively; (**b**) *Bolbosoma capitatum*, scale bars 2 and 5 cm respectively; (**c**) *Caligus elongatus*, scale bars 1 mm, 5 and 5 cm respectively.

We adapted the method initially proposed by^47^ to determine the individual mass of ciliates. We modelled with commercial air-drying clay the body of the selected specimens of *C*. *oblonga, B*. *capitatum* and *Ca. elongatus* to approximate scales of 16–19, 2–9 and 26–39, and the appendages of *Ca. elongatus* to 92–217 times larger than the real structures, respectively (Figs 2, 3). Then, we measured the volume of water displaced by each model in graduated cylinders to the nearest 0.05 ml for *C*. *oblonga* and 0.5 ml for *B*. *capitatum* and *Ca. elongatus*, respectively. The volume of the specimen was calculated as (Eq. 2):

2 $${V}_{s}={V}_{m}\,\ast \,{({L}_{s}/{L}_{m})}^{3}$$

Where *Vm* and *Lm* are the clay model volume and length respectively; and *Vs* and *Ls* are the specimen’s volume and length respectively.

### Indirect Method 2: Extracting Mass From Images

Image analysis is a suitable tool to indirectly estimate biomass because it is non-destructive, time- and cost-effective (Appendix S1) and allows continuous observation of individual development^48^. Estimating mass of single individuals from images is a common concern in distant disciplines of biology and different solutions have arisen (e. g. aquaculture^49^; zooplanktology^29,31^; palaeontology^50^; or botany^48^).

The indirect method 2 was divided into 3 submethods according to the morphology of the transversal sections of the individuals understudy (Fig. 1).

Area By Depth By Density (Flat Section): This method was applied to *C*. *oblonga* and is based on Lambden & Johnson^21^. These authors squashed specimens in a microwell of known depth to obtain the ventral area of the organism by means of image acquisition and analysis software. Individual volume was then estimated as the product of microwell depth by the ventral area and converted into biomass after multiplying by tissue density. We drew in ventral view the outline of the body, pharynx, and ventral sucker of the 20 selected individuals under a microscope fitted with a drawing tube (Nikon Optiphot-2 at 10× magnification). Drawings were scanned at 600 ppi and were saved in TIFF format. We measured the area (µm^2^) of individuals in ventral view with Fiji-ImageJ version 1.51n^51^. As our specimens were mounted on permanent slides, their depth^21^ was the mean thickness of each individual (measured as indicated above). To obtain individual body volume, we multiplied body area by the mean thickness of each individual (Fig. 1a). The advantage of this approach compared with that of Lambden & Johnson^21^ is that it can be applied to both fresh and permanent mounted material. Additionally, using the same approach, we added the volume of the ventral sucker to the body mass of each individual.

**Figure 4:**
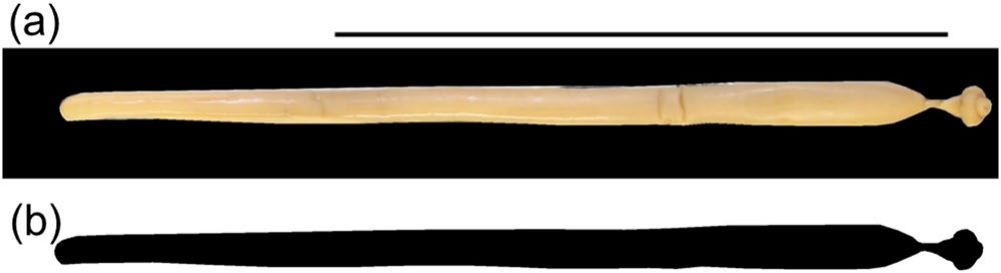
Two steps in image analysis process to estimate individual mass from images as volume of revolution. (**a**) *Bolbosoma capitatum* picture with Feret diameter aligned with image wide margin (i.e. angle minimised); (**b**) binary conversion of the specimen after thresholding, white pixels represent the background and black pixels the animal surface. Scale bar 5 cm.

Volume Of Revolution By Density (Subcircular Section): In *B*. *capitatum*, we photographed the 20 selected individuals with a digital camera (Canon EOS 700D EFS 15–85 mm) held by a camera stand (Kaiser RSX). Pictures were taken at 5184 dpi. By means of GIMP version 2.8.18^52^, we extracted the individual from the picture and placed it on a black background (Fig. 4a). Using ImageJ, pictures were scaled to convert linear measurements into µm. Then, pictures were thresholded to make them binary (i.e. tell apart object pixels from background pixels) (Fig. 4b) and rotated to render the Feret diameter of the object horizontal. Images were then saved as text image (Appendix S2). After thresholding, ImageJ saves object pixels as 255 and background pixels as 0. Lastly, we processed text images with a R^53^ script (Appendix S3). Parameters included in the script were:

ratio: µm px^−1^

- As the script initially expressed the body volume in pixels^3^, we converted body volume into µm^3^ using the scale computed above with ImageJ.
- Based on the text image, each column of object pixels was treated as a one-pixel-wide slice (i.e. transversal section) with a regular circular shape. Thus, the volume of each individual was computed as the sum of volumes of each slide.

rho: tissue density as g ml^−^

- To calculate body mass, volume was multiplied by the estimated tissue density to obtain body mass in mg.

Complex Morphologies (combining flat and subcircular sections): To deal with more complex morphologies, as in *Ca. elongatus* (Fig. 1c), we processed each specimen dividing the body into portions according to (i) their transversal section (flat vs subcircular) and (ii) when flat, according to similar mean thickness. In *Ca. elongatus*, the body can be easily divided as per (i) into main body and appendages (flat sections), and egg strings (subcircular section). Following (ii), three large body areas with similar mean thickness, were recognized: the cephalothorax, the fourth pedigerous somite and the genital-abdominal complex. Additionally, we measured the area of one appendage of each of its 8 pairs (Tables S2 and S3). We photographed the 20 selected individuals with a Nikon Fotomicroscope E800 at 4× magnification to obtain the body surface and at 10× to obtain the surfaces of appendages. Pictures were taken at 5184 dpi.

To estimate volumes of main body areas and appendages (i.e. flat section pieces) we applied the method described in section (2.1), analogously to *C. oblonga*. For egg strings (i.e. subcircular section), we used the method explained in section (2.2), analogously to *B*. *capitatum*. Finally, volumes of pieces were added up.

### Indirect Method 3: Approximation To Regular Geometric Shapes

**Figure 5:**
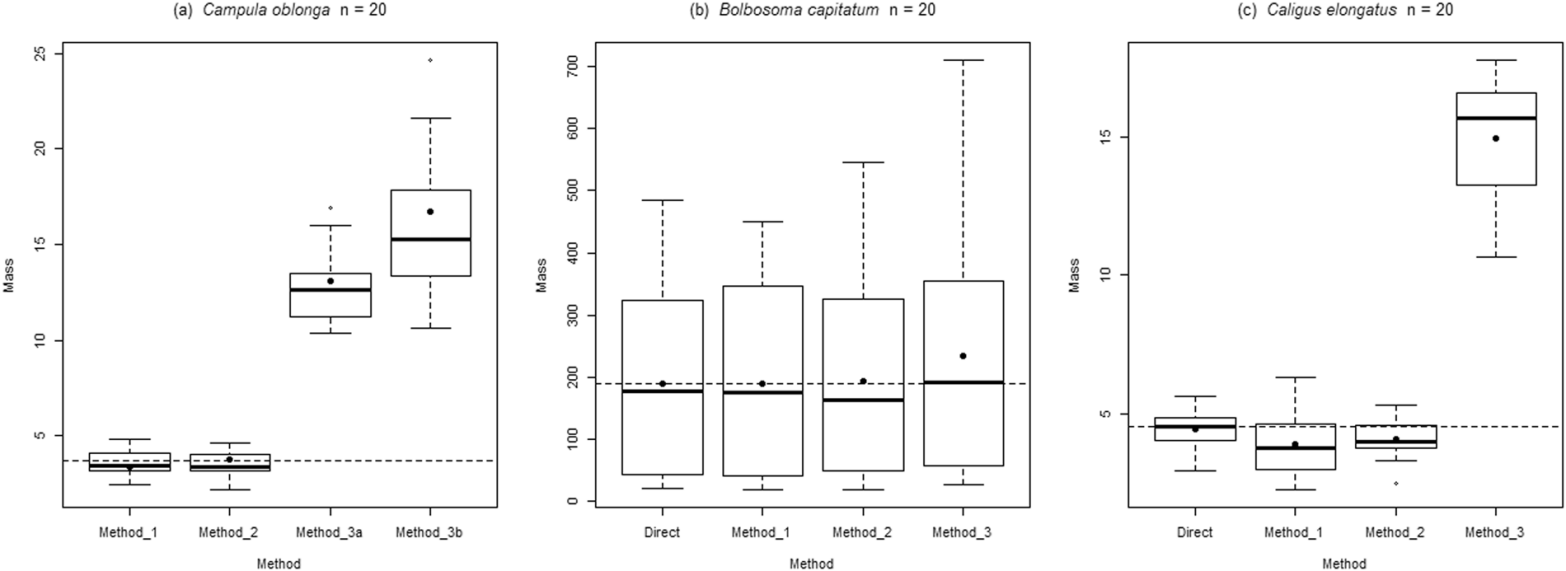
Boxplot of mass (mg) estimated by different methods for (**a**) *Campula oblonga*, (**b**) *Bolbosoma capitatum* and (**c**) *Caligus elongatus*. Continuous line indicates the median value for each method. Fill point represents the mean individual mass for each method. Dashed line represents the mean mass value of a single individual obtained by direct method. Method 1: clay model; Method 2: image analysis; Method 3: geometric approximation, 3a ellipsoid, 3b cylinder.

We measured maximum body length (BL) and width (BW) of individuals by approximating body volume to simple geometric shapes (e.g.)^17,38^. In *C*. *oblonga*, body volume was approximated to an ellipsoid (e.g.)^30,32^ (Method 3a in Fig. 5 and Table S4) and to a cylinder (e.g.)^28,38^ (Method 3b in Fig. 5 and Table S4). For *B*. *capitatum*, body volume was calculated assuming a cylindrical shape (e.g.)^28,30,35^. In *Ca. elongatus*, body volume was approximated to an ellipsoid (e.g.)^29^ and egg strings to a cylinder. In the three model organisms, we based our measurements on total BL, maximum BW and body depth equal to BW (Fig. 1).

### Statistic Analyses

Due to the nature of our samples, we performed two kinds of statistical analyses to test for differences between the estimates obtained directly and those computed indirectly. In *C*. *oblonga*, we compared the average individual mass obtained directly for a sample of individuals with the corresponding mean weight obtained for each individual with each indirect method (i.e. individuals mounted on permanent slides) (methods 1, 2, 3a and 3b) using *t-*tests for one sample with Bonferroni correction (i.e. alpha = 0.05/4). In *B*. *capitatum* and *Ca. elongatus*, we used Linear Mixed Effect Models to compare the different methods (fixed factor) across individual specimens (random factor). All statistical analyses were carried out with R packages lme4^54^ and stats^53^.

### Data Availability

Collection of the Marine Zoology Unit, Cavanilles Institute of Biodiversity and Evolutionary Biology, Science Park, University of Valencia. Accession numbers of samples: *Campula oblonga* mounted specimens: CN491122, CN491158, CN610121, CN610128, CN610140, CN675077, CN677015, CN677090-92, CN677117, CN677120, CN680012, CN681007, CN687088, CN687094, CN696110, CN696123, CN716016-17; weighed specimens: CN707, CN716; *Bolbosoma capitatum*: 04013, 04150, 04192, 04196, 04199, 04202, 04209, 04299, 04305, 08823, 08826, 08830; *Caligus elongatus*: CT1E035, CT2C039, CT2C051, CT3B017, CT3B035, CT3B037. ImageJ and R scripts can be downloaded as online supporting information (Appendix S2 and S3). All data analysed during this study is included in this article (Tables S1–6).

## Results

### Tissue Densities

The estimated tissue densities (± absolute errors) were 1.06 ± 0.03 g ml^−^ for *C. oblonga*, 1.05 ± 0.10 g ml^−^ for *B. capitatum* and 1.15 ± 0.01 g ml^−^ for *Ca. elongatus*.

### Thickness Estimation

Mean body and mean thickness of appendages (±standard error) of the species are shown in Tables S1–3.

### Estimations of Masses

Individual mass estimated for each specimen is presented in Tables S4, S5 and S6 for *C. oblonga, B. capitatum* and *Ca. elongatus* respectively. A comparison of the accuracy of the methodologies is provided in Fig. 5. For *C*. *oblonga*, the average individual mass estimated by indirect methods 1 and 2 were very similar to and not significantly different from that estimated with the direct method (*t* = −1.86, *P = *0.08; *t* = 0.74, *P = *0.47;). In contrast, means obtained by methods 3a and 3b differed significantly from that computed directly (*t* = 20.02, *P < *0.001; *t* = 13.73, *P < *0.001). These methods overestimated mean individual mass by 3.5 to 4.4 times. For *B*. *capitatum* and *Ca. elongatus*, the mean values obtained with methods 1 and 2 were very close to and not significantly different from those of the direct method (Table 1). In contrast, method 3 showed a higher and significantly different value of mean individual mass (Table 1).

**Table 1:** Linear Mixed Model analyses between different methods for individual mass estimation of *Bolbosoma capitatum* and *Caligus elongatus*.

	Estimate	Std. Error	t value	P value
*Bolbosoma capitatum*				
Intercept	0.19	0.04	4.74	0.00
Method 1	0.00	0.01	0.29	0.77
Method 2	0.00	0.01	0.03	0.97
Method 3	0.05	0.01	3.51	0.00
*Caligus elongatus*				
Intercept	4.43	0.30	14.77	0.00
Method 1	−0.53	0.37	−1.44	0.15
Method 2	−0.36	0.37	−0.99	0.32
Method 3	10.50	0.37	28.65	0.00

## Discussion

In this paper, we evaluated the accuracy of indirect methods estimating individual parasite mass. Our results showed that the indirect methods proposed herein provided the closest approximation to the direct estimation of average individual mass. Despite the extensive use of approximation to geometrical shapes (e.g.)^28–30,34^, method 3 was far from satisfactory in all situations as it grossly overestimates biomass.

Regarding species tissue densities, although they are available in the literature (e.g.)^17^, we decided to measure density independently as additional validation of our biomass measurements. Our density results agree with that published previously for adult flatworms^17^ (1.1 g ml^−^) and crustaceans^55^ (1.098–1.506 g ml^−^). According with this, we can assume that our specimens were fully rehydrated. However, it is worth saying that if a researcher wanted to know the tissue density of a species from stored specimens, they would check the completely rehydration of the specimens.

Classical approaches to estimate biomass of small invertebrates have relied on approximations to regular geometric shapes, in most cases cylinders or ellipsoids^28–30,38,56^. However, these regular geometric structures might be quite different from the real morphology of organisms^31^ and this could often lead to misinterpretations. Particularly, when extrapolating biomass results to community and/or ecosystem studies, the effect of these biases can be additive. As shown in Fig. 5, classical approaches (i.e. indirect method 3) provided estimators well over the reference values. Furthermore, when assuming a regular geometric shape, the contribution of salient structures, for example ventral sucker of flatworms and paired appendages of crustaceans in our case, or tail of cercaria (e.g.)^35^, or expansions of the tegument of molluscs (e.g.)^18^, among others, is neglected. Furthermore, we would also like to emphasise that if a researcher plans to estimate the biomass a population using any of the methods proposed in this paper: (1) they should consider the phenotypic variability of their population (identifying if required morphological categories according to life stage, sex etc.), (2) estimate the mean weight of a representative number of organisms of each category and (3) multiply the mean weight of an individual of a category to the observed proportion of the category in the population.

Comparing the indirect method 1 (modified from)^47^ with 2, both require estimation of body thickness and yielded similar results. Both approaches are time- and cost-effective and easy to apply in most situations. In addition, they are non-destructive and the new estimations of individual mass from images are based on open-source software. Note also that the boxplots shown in Fig. 5 convey the variation of the sample for each method, which results from the inherent sample variance ± the measurement error. This facilitates assessment of the measurement error between methods. Overall the error committed in methods 1 and 2 seem fairly similar to each other and to those incurred in the direct estimation of weights. The exception is apparent larger variation associated to method 1 when applied to *Ca. elongatus*. As this species was the most morphologically complex, this observation suggests that measurement error is probably dependent of species shape and skill of the modeler. So, although for more morphologically complex organisms, clay modelling (i.e. method 1) could be the best option, it may require the intervention of a qualified artist to render realistic representations of model organisms, thereby minimizing measurement error. In any case the average value of the biomass estimator of *Ca. elongates* obtained was not significantly different from those obtained directly or applying method 2.

Method 2.2 would work best with straight and symmetrical organisms with convex contours. For asymmetric and/or extremely appendage-ornamented organisms, one-pixel thick slices will not add-up correctly, leading to overestimation of individual mass. Nonetheless, the inaccuracy for estimating mass of complex morphologies can be solved by dividing the specimens into parts as demonstrated herein with the crustacean model species (method 2.3). We would like to highlight the importance of scanning images at high resolution to minimise the error associated to image acquisition.

To fill the gap of invertebrate descriptions^46^, we developed an easy technique to measure thickness of mounted individuals using a light microscope, the commonest way to study morphology of small invertebrates. We foresee that our thickness estimator will be very useful to measure thickness of any kind of small invertebrate (e.g. plankton or soil-dwelling species) or structures on a slide. There are three main advantages of our method: (1) It allows estimating thickness of organisms previously stored in collections as it can be applied to specimens on permanent and non-permanent mounts. (2) The specimens can be recovered after measuring and used in further applications. (3) In comparison to Lambden & Johnson^21^ estimation of body thickness, our measurement can be applied to specimens thicker than 0.127 mm, which cannot be squashed into a plate. Additionally, our estimation of mass from images is less expensive as the use of a special plate is not required. Novack-Gottshall^18^ found that the ATD method (i.e. the product of lengths of the three major axes of invertebrate fossil bodies) was the best predictor of body volume as representative of body mass. Thus, Lambden & Johnson^21^ and our 2.1 indirect method proposed represent similar strategies to estimate mass of small invertebrate individuals, but more elaborated than that of Novack-Gottshall^18^. Lagrue & Poulin^35^ measured thickness of specimens placing them in lateral view under a stereomicroscope. Although this approach is straightforward, it might be tedious and inaccurate to apply to very thin and/or small invertebrates. A limitation of method 2.1 lies in the availability of material to measure thickness. However, this could be solved by measuring thickness from morphologically similar species.

## Conclusion

Estimating biomass of small invertebrates poses a series of challenges that can be overcome by using indirect methods that have been rarely tested for accuracy. Our study shows that the indirect methods proposed in this paper provide a good approximation to the real body mass and are much more accurate than approximating body morphology to regular geometric figures, as previously applied to small invertebrates in the literature. In particular, our method for estimating biomass from images seems more time- and cost-effective than previous approaches, catering for the growing need of obtaining reliable estimates of invertebrate biomass^48^. We validated the shaping methodology originally described for unicellular ciliates^47^ to be generally applied to small invertebrates. This clay shaping-based method may be particularly valuable for organisms with complex morphology, although with the cost of time and skills investment, that may render this approach only useful for model species. The benefit of our proposed methods is threefold. They allow recovering the material after use, can be applied to both fresh and mounted specimens on permanent slides and the images and figurines generated can be permanently archived and used in further studies.

## Electronic supplementary material

Supplementary material
