# Author Correction: Evaluation of three methods for biomass estimation in small invertebrates, using three large disparate parasite species as model organisms

**DOI:** 10.1038/s41598-018-26011-5

**Published:** 2018-05-31

**Authors:** Cristina Llopis-Belenguer, Isabel Blasco-Costa, Juan Antonio Balbuena

**Affiliations:** 1 Symbiosis Lab, Cavanilles Institute of Biodiversity and Evolutionary Biology, University of Valencia, PO Box 22085, 46071 Valencia, Spain; 2 Natural History Museum of Geneva, PO Box 6434, CH-1211 Geneva 6, Switzerland

Correction to: *Scientific Reports* 10.1038/s41598-018-22304-x, published online 01 March 2018

This Article contains errors.

In the legend of Figure 1,

“Phenotypic traits that justify the use of the model species. (a) *Campula oblonga*, (b) *Bolbosoma capitatum* and (c) *Caligus elongatus* as a model species of the biomass indirect estimation methods. Scale bars 0.5 mm, 2 cm and 5 cm respectively.”

should read:

“Phenotypic traits that justify the use of the model species. (a) *Campula oblonga*, (b) *Bolbosoma capitatum* and (c) *Caligus elongatus* as model species of the biomass indirect estimation methods. Scale bars 2, 20 and 1 mm, respectively.”

Additionally, in the legend of Figure 3

“Specimens and clay figurines of the model species. (a) *Campula oblonga*, scale bars 0.5 mm and 2 cm respectively; (b) *Bolbosoma capitatum*, scale bars 2 and 5 cm respectively; (c) *Caligus elongatus*, scale bars 1 mm, 5 and 5 cm respectively.”

should read:

“Specimens and clay figurines of the model species. (a) *Campula oblonga*, scale bars 2 and 20 mm, respectively; (b) *Bolbosoma capitatum*, scale bars 20 and 50 mm, respectively; (c) *Caligus elongatus*, scale bars 1, 50 and 50 mm, respectively.”

Finally, in the Materials and Methods section,

“Area By Depth By Density (Flat Section)”

should read:

“Area By Depth By Density (Flat Section) (Indirect Method 2.1)

“Volume Of Revolution By Density (Subcircular Section)”

should read:

“Volume Of Revolution By Density (Subcircular Section) (Indirect Method 2.2)”

“Complex Morphologies (combining flat and subcircular sections)”

should read:

“Complex Morphologies (combining flat and subcircular sections) (Indirect Method 2.3)”

