# Supplementary material for: Evaluation of three methods for biomass estimation in small invertebrates, using three large disparate parasite species as model organisms
Source: Sci Rep. 2018 Mar 1;8:3897. doi: 10.1038/s41598-018-22304-x (PMC5832816; doi:10.1038/s41598-018-22304-x)
Supplement: Supplementary file 1 — Supplementary material [file 41598_2018_22304_MOESM1_ESM.pdf]

Evaluation of three methods for biomass estimation in small invertebrates, using three large disparate parasite species as model organisms

Cristina Llopis-Belenguer[1*], Isabel Blasco-Costa[2] & Juan Antonio Balbuena[1]

[1]Symbiosis Lab, Cavanilles Institute of Biodiversity and Evolutionary Biology, University of Valencia, PO Box 22085. 46071 Valencia, Spain

[2]Natural History Museum of Geneva, PO Box 6434, CH-1211 Geneva 6, Switzerland

* Correspondence author: cristina.llopis.belenguer@gmail.com

Table S1. Thickness (µm) of *Campula oblonga* specimens at pharynx level, ventral sucker level, posterior end of vitellarium and thickness of the ventral sucker.

	Pharynx	VS level	Posterior end	VS
Co_1	484	465	435	254
Co_2	517	342	362	230
Co_3	435	414	383	200
Co_4	460	378	394	236
Co_5	399	403	394	204
Co_6	494	427	471	212
Co_7	412	352	339	297
Co_8	352	424	399	241
Co_9	438	318	342	261
Co_10	452	297	404	266
Co_11	317	207	370	228
Co_12	383	234	388	266
Co_13	457	255	406	241
Co_14	444	354	404	207
Co_15	278	311	411	258
Co_16	437	373	407	219
Co_17	351	287	366	210
Co_18	417	432	434	194
Co_19	414	438	389	166
Mean	419	350	392	231
SD	59	74	33	31

VS: ventral sucker.

Table S2. Thickness (µm) of *Caligus elongatus* specimens at lateral area of the cephalothorax, cephalothorax, fourth pedigerous somite, genital segment and abdomen.

	Lateral (cephalotorax)	Thorax (cephalotorax)	Fourth pedigerous somite	Genital segment	Abdomen
Ce_1	452	729	231	345	266
Ce_2	398	588	297	463	192
Ce_3	327	549	280	336	164
Ce_4	428	592	192	497	246
Ce_5	306	484	296	463	292
Ce_6	283	490	178	521	258
Ce_7	223	357	152	423	213
Ce_8	358	484	280	444	250
Ce_9	445	687	275	466	385
Ce_10	444	662	219	374	212
Ce_11	400	651	232	482	237
Ce_12	234	448	221	437	258
Ce_13	278	562	210	332	308
Ce_14	323	444	209	438	259
Ce_15	337	447	182	306	201
Ce_16	379	543	192	388	118
Ce_17	237	278	157	222	293
Ce_18	287	460	253	305	240
Ce_19	333	518	226	302	181
Ce_20	348	639	281	305	259
Mean	341	531	228	392	242
SD	72	113	45	82	57

Table S3. Thickness (µm) of appendages of *Caligus elongatus*.

	1st antenna	2nd antenna	Maxilla	Maxilliped	1st leg	2nd leg	3rd leg	4th leg
Ce_1	74	157	160	149	185	115	116	201
Ce_2	83	133	110	157	155	152	129	144
Ce_3	118	168	129	44	165	141	154	132
Ce_4	49	154	104	138	129	115	64	93
Ce_5	113	146	94	181	148	165	74	140
Ce_6	82	110	126	144	143	174	71	119
Ce_7	113	129	96	130	171	107	80	102
Ce_8	126	122	96	149	104	96	63	105
Ce_9	110	82	116	143	140	173	155	118
Ce_10	89	108	108	141	132	110	89	148
Ce_11	94	94	97	146	115	126	86	148
Ce_12	91	126	88	144	113	151	130	141
Ce_13	122	111	89	162	144	137	115	144
Ce_14	110	91	97	135	121	127	119	126
Ce_15	94	124	83	129	121	121	100	133
Ce_16	122	135	82	108	96	111	105	135
Ce_17	88	83	86	132	99	111	102	99
Ce_18	113	116	91	129	99	105	107	93
Ce_19	108	107	104	165	127	129	138	121
Ce_20	110	129	121	108	149	137	143	138
Mean	101	121	104	137	133	130	107	129
SD	19	24	19	28	25	23	29	25

Table S4. Individual mass (mg) of specimens of *Campula oblonga*. Note that after obtaining body volume for each method, it was multiplied by tissue density.

INDIVIDUAL (BL=6532 µm; BW=1748 µm)	Direct	Method 1	Method 2	Method 3a	Method 3b
n = 20	3.43				
n = 21	3.95				
Co_1		4.50	3.89	12.69	13.98
Co_2		4.32	4.54	15.18	18.87
Co_3		4.04	3.58	12.26	14.52
Co_4		3.43	3.12	10.85	12.20
Co_5		3.17	3.09	10.53	10.63
Co_6		4.61	4.13	11.67	15.34
Co_7		3.74	4.19	12.61	17.63
Co_8		4.49	3.98	14.65	15.87
Co_9		3.48	3.42	12.30	13.37
Co_10		3.28	2.79	10.73	11.78
Co_11		2.44	2.17	10.38	13.52
Co_12		3.15	3.37	13.17	16.75
Co_13		3.45	2.92	12.92	15.18
Co_14		3.80	3.11	12.79	18.09
Co_15		3.25	2.53	13.41	18.80
Co_16		4.63	3.27	16.90	21.59
Co_17		3.34	3.39	13.53	16.81
Co_18		4.83	4.02	16.02	24.65
Co_19		5.16	4.34	17.81	27.89
Co_20		3.08	2.35	11.35	16.29
MEAN	3.69	3.81	3.41	13.09	16.69
SD	0.37	0.72	0.67	2.10	4.25

BL and *BW*: body length and width respectively; *n* number of individuals; Method 1: clay model; Method 2: image analysis; Method 3: geometric assumption: 3a ellipsoid. 3b cylinder.

Table S5. Individual mass (mg) of specimens of *Bolbosoma capitatum*. Note that after obtaining body volume for each method, it was multiplied by tissue density.

INDIVIDUAL (BL = 59268 µm; BW = 1904 µm)	Host species	Direct	Method 1	Method 2	Method 3
Bc_1	Gm	183	173	196	191
Bc_2	Gm	205	178	186	213
Bc_3	Gm	216	205	184	220
Bc_4	Gm	187	160	165	191
Bc_5	Gm	172	165	251	227
Bc_6	Pc	312	331	343	349
Bc_7	Pc	426	436	445	544
Bc_8	Pc	386	397	450	474
Bc_9	Pc	336	320	349	359
Bc_10	Pc	445	545	415	711
Bc_11	Pc	485	434	438	705
Bc_12	Gm	85	88	84	101
Bc_13	Gm	58	50	65	64
Bc_14	Gm	36	41	39	53
Bc_15	Gm	43	47	38	45
Bc_16	Gm	69	79	77	77
Bc_17	Gm	41	45	37	57
Bc_18	Gm	19	17	18	25
Bc_19	Gm	42	48	42	55
Bc_20	Gm	22	18	22	26
MEAN		188	189	192	234
SD		158	164	160	221

BL and *BW*: body length and width respectively; *n* number of individuals; Method 1: clay model; Method 2: image analysis; Method 3: geometric assumption. Gm *Globicephala melas*; Pp *Pseudorca crassidens*.

Table S6. Individual mass (mg) of specimens of *Caligus elongatus*. Note that after obtaining body volume for each method, it was multiplied by tissue density.

INDIVIDUAL BL = 5620 µm; BW = 2321 µm	Direct	Method 1	Method 2	Method 3
Ce_1	4.60	3.80	4.94	14.50
Ce_2	4.93	3.28	4.67	15.93
Ce_3	4.97	3.58	3.97	15.13
Ce_4	4.53	2.74	4.15	11.67
Ce_5	4.50	5.63	4.00	15.46
Ce_6	3.97	2.85	3.72	10.66
Ce_7	4.50	2.24	3.29	17.50
Ce_8	4.33	2.52	3.76	11.11
Ce_9	3.95	6.31	4.68	12.26
Ce_10	4.65	5.44	4.66	16.69
Ce_11	5.00	4.08	5.31	16.44
Ce_12	5.65	5.61	4.35	17.39
Ce_13	4.10	2.50	3.95	16.28
Ce_14	5.33	4.86	4.29	17.77
Ce_15	4.53	3.83	3.85	16.80
Ce_16	4.77	4.26	4.46	16.52
Ce_17	2.93	3.20	2.47	12.71
Ce_18	3.57	3.09	3.38	13.89
Ce_19	3.67	3.75	3.54	16.14
Ce_20	4.07	4.43	3.84	13.79
MEAN	4.43	3.90	4.06	14.93
SD	0.33	1.17	0.65	2.23

BL and *BW*: body length and width respectively; *n* number of individuals; Method 1: clay model; Method 2: image analysis; Method 3: geometric assumption.

Appendix S1. Relative costs and time required of the indirect methods reviewed.

When working on biomass of invertebrates, the final method applied mainly depends on the aim or the requirements of precision of the study, the technical support of laboratory and the cost of the method[1]. The common physicochemical indirect methods (e.g. wet, dry, ash-free dry, protein and carbon weights) have high costs (i.e. thousand €) and time (i.e. days) associated[1, 2]. Thus, these methods may be unaffordable for scientists with time or budgetary limitations[3]. In this Appendix, we aim to present the table below (Table appendix S1) of the relative costs and time required of the indirect methods reviewed: clay modelling, extracting mass from images and geometric approximation. As initial steps of three methods require the same laboratory equipment and consumables we have not considered such costs in the table of comparisons. So, depending on the studied species, laboratory equipment required will be a microscope gearing a camera or a drawing tube or a digital camera held by a camera stand. For the consumables, the three methods need for saline solution, petri dish, slide and cover, tweezers, graduated cylinder and scale.

Table appendix S1. Relative costs and time required of the indirect methods reviewed.

	Clay modelling	Image analysis	Geometric approximation
Price/ individual assessed	Very low (Clay and a ruler are needed)	Low (A computer and free software are required)	Low (A computer and free software are required)
Time/ individual assessed*	High (1 to 1.5 workdays/ individual assessed)	Medium (5 hours/ individual assessed)	Low (30 minutes to 1 hour/ individual assessed)
Precision (see Results section)	High	High	Very low

* The “time/ individual assessed” factor rely on the complexity of the morphology of the species assessed. We have considered the most complex species in our study (i.e. *Caligus elongatus*).

References in Appendix S1

1. Postel, L., Fock, H. & Hagen, W. in *ICES Zooplankton Methodology Manual* (eds Harris, R., Wiebe, P., Lenz, J., Skjoldal, H. R. & Huntley, M.) 83-192 (Academic Press, London, 2000).
2. Sandak, A., Sandak, J., Waliszewska, B., Zborowska, M. & Mleczek, M. Selection of optimal conversion path for willow biomass assisted by near infrared spectroscopy. *iForest - Biogeosciences and Forestry* **10**, 506 (2017).
3. Meyer, C. K., Peterson, S. D. & Whiles, M. R. Quantitative Assessment of Yield, Precision, and Cost-Effectiveness of Three Wetland Invertebrate Sampling Techniques. *Wetlands (Wilmington)* **31**, 101-112 (2011).

Appendix S2. ImageJ script for extracting individuals from images.

```
open() //To select and open the input image file
//Step 1
        run("Set Scale...");
//Step 2
        run("8-bit");
//Step 3
        setAutoThreshold("Default dark");
        // Alternatively, the user may threshold the image
        // interactively by running the next two lines
        //run("Threshold...");
        //setOption("BlackBackground", true);
//Step 4
        run("Convert to Mask");
//Step 5
        run("Analyze Particles...", "display");
//Step 6
        run("Rotate... ");//Rotate to Feret angle
                          // obtained in Results Step 5
run("Make Binary");
run("Auto Crop (guess background color)");
//Step 7
saveAs("Text Image...");
```

```
 // Step 1
 run("Set Scale...");
```

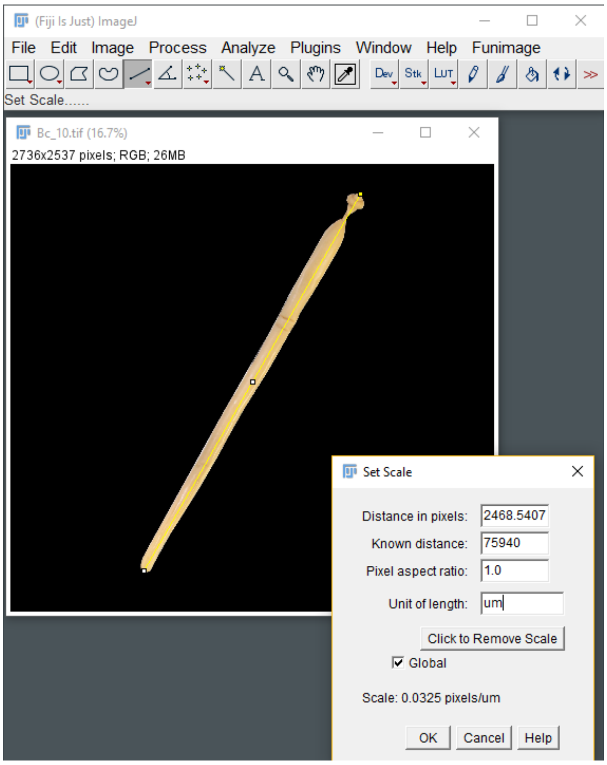

89

```
// Step 2
run("8-bit");
```

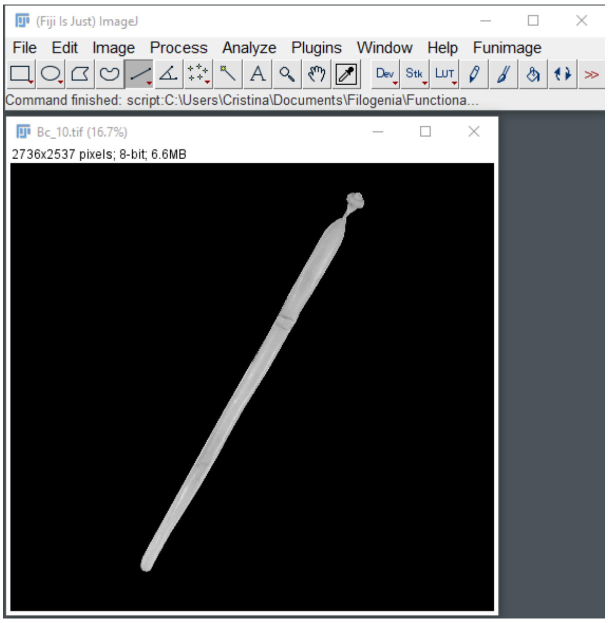


```
// Step 3
setAutoThreshold("Default dark");
```

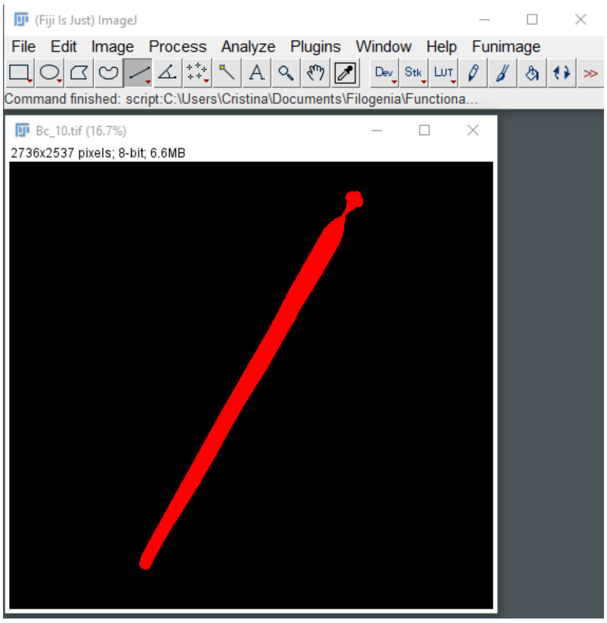

90

```
// Step 4
run("Convert to Mask");
```

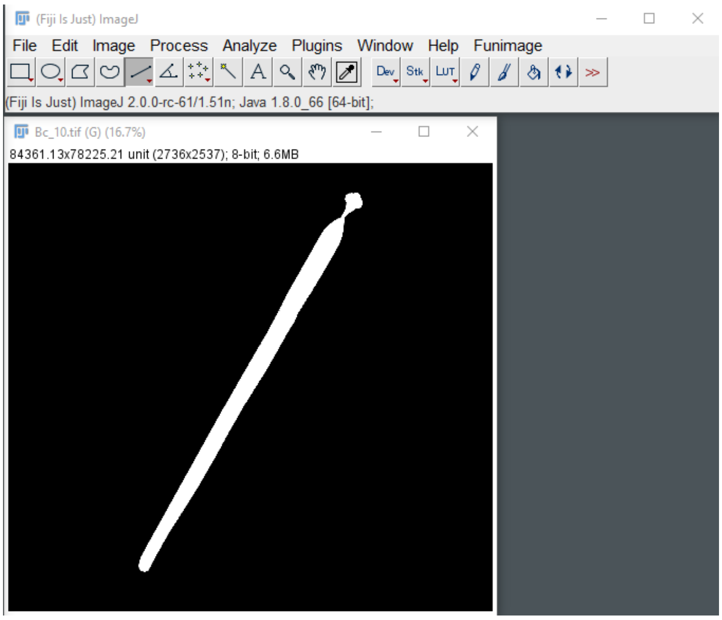


```
// Step 5
run("Analyze Particles...", "display");
```

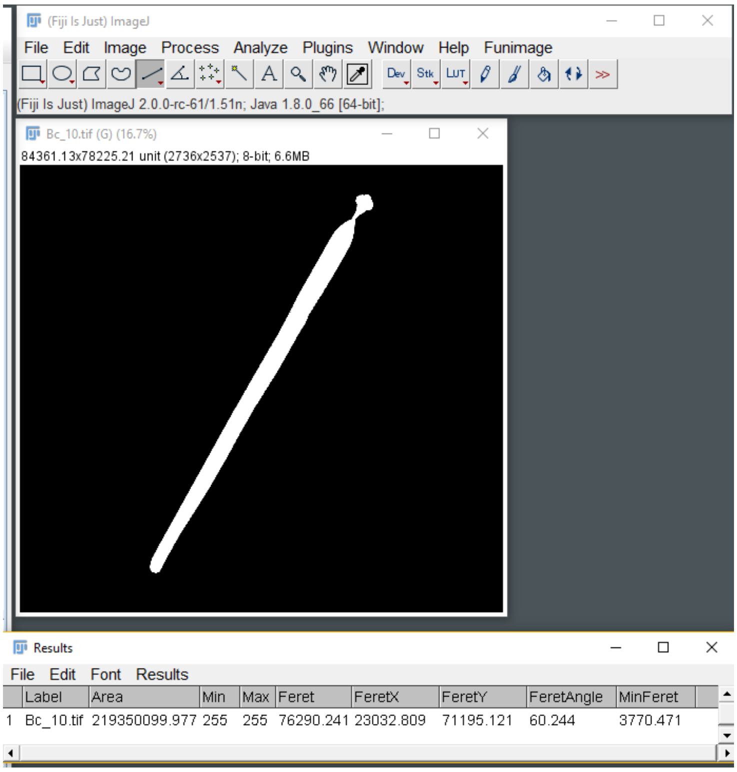

```
// Step 6
run(""Rotate... ");
run("Make Binary");
```

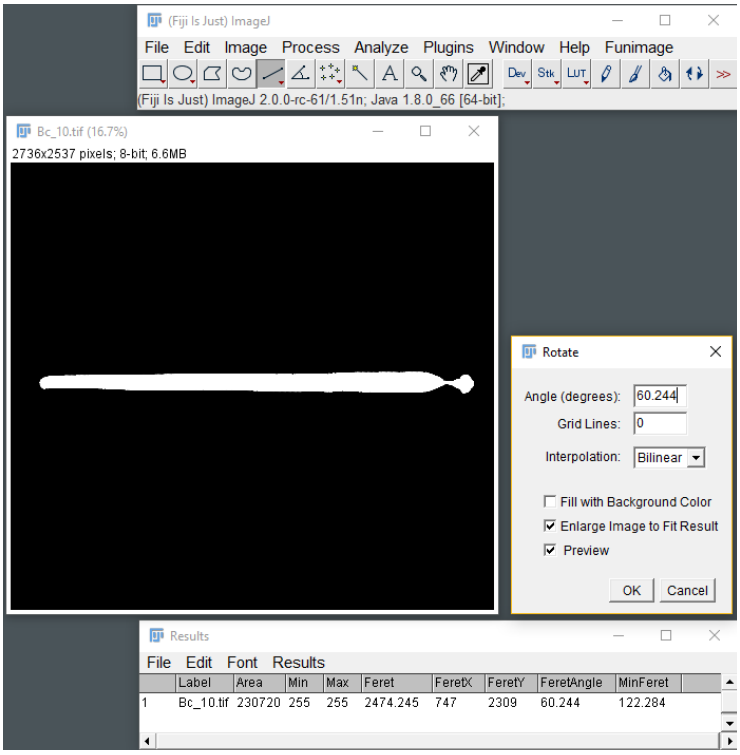


```
// Step 7
run("Auto Crop (guess background color)");
saveAs("Text Image...");
```

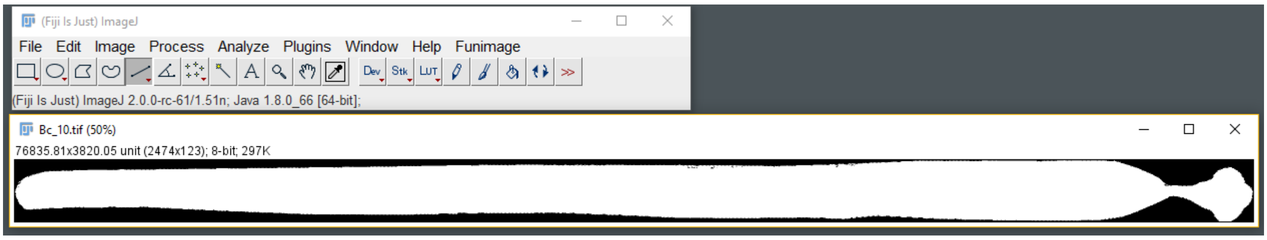

```
#Appendix S3 R script for estimating mass of an individual with a circular
transversal section as volume of revolution.
# It reads text images and computes their revolution volume
# The script is intended for inputs in µm (length) and g/ml (tissue density)
# and computes the specimen's mass in mg
#
################## SCRIPT STARTS HERE #
#Read images and compute their revolution volume

ratio = 0.0119 # pixels/µm -- scale obtained with ImageJ (Set Scale)
rho = 1.05 # g/ml -- tissue density
bin.image <- as.matrix(read.table(file.choose())) # select text image to
analyze interactively

#read image as matrix and convert to binary matrix
vol.image <- function (x, sc, dens) {
x <- x/255 # sets matrix to binary
if (all(x %in% 0:1)==FALSE) {stop("The image matrix is not binary")} else {
x <- x[which(rowSums(x) != 0), which(colSums(x) != 0)] #crop image
width <- colSums(x)
sc <- 1/sc # invert scale to transform pixels into µm
M <- pi*sum((width/2)**2) * sc**3 * dens
M <- M*1e-9 # returns body mass in mg
}
return (M)
}

body_mass <- vol.image (bin.image, ratio, rho)

print(paste("The specimen's mass is ", round(body_mass,2), " mg"))
```
